# Do older surgeons have safer hands? A retrospective cohort study

**DOI:** 10.1186/s13019-022-01943-2

**Published:** 2022-09-01

**Authors:** Victoria Rizzo, Edward J. Caruana, Kathrin Freystaetter, Gareth Parry, Stephen C. Clark

**Affiliations:** 1 Cardiothoracic Centre, Freeman Hospital, Newcastle upon Tyne, NE7 7DN United Kingdom; 2 Department of Thoracic Surgery, Glenfield Hospital, Leicester, LE3 9QP United Kingdom; 3 Northumbria University, Newcastle upon Tyne, Tyne and Wear, NE1 8ST United Kingdom; 4 Guy’s and St Thomas’ NHS Foundation Trust, Westminster Bridge Road, London, SE1 7EH United Kingdom

**Keywords:** Surgery, Patient safety, Medical education, Human factors, Hospital medicine

## Abstract

**Background:**

For complex surgical procedures a volume-outcome relationship can often be demonstrated implicating multiple factors at a unit and surgeon specific level. This study aims to investigate this phenomenon in lung transplantation over a 30-year period with particular reference to surgeon age and experience, cumulative unit activity and time/day of transplant.

**Methods:**

Prospective databases identified adult patients undergoing isolated lung transplantation at a single UK centre between June 1987 and October 2017. Mortality data was acquired from NHS Spine. Individual surgeon demographics were obtained from the General Medical Council. Student *t*-test, Pearson’s Chi-squared, Logistic Regression, and Kaplan–Meier Survival analyses were performed using Analyse-it package for MicrosoftExcel and STATA/IC.

**Results:**

954 transplants (55.9% male, age 44.4 ± 13.8 years, 67.9% bilateral lung) were performed, with a median survival to follow-up of 4.37 years. There was no difference in survival by recipient gender (*p* = 0.661), between individual surgeons (*p* = 0.224), or between weekday/weekend procedures (*p* = 0.327).

Increasing centre experience with lung transplantation (OR1.001, 95%CI: 1.000–1.001, *p* = 0.03) and successive calendar years (OR1.028, 95%CI: 1.005–1.052, *p* = 0.017) was associated with improved 5-year survival.

Advancing surgeon age at the time of transplant (mean, 48.8 ± 6.6 years) was associated with improved 30-day survival (OR1.062, 95%CI: 1.019 to1.106, *p* = 0.003), which persisted 5 years post-transplant (OR1.043, 95%CI: 1.014–1.073, *p* = 0.003).

Individual surgeon experience, measured by the number of previous lung transplants performed, showed a trend towards improved outcomes at 30 days (*p* = 0.0413) with no difference in 5-year survival (*p* = 0.192).

**Conclusions:**

Our study demonstrates a relationship between unit volume, increasing surgeon age and survival after lung transplantation. A transplant volume: outcome relationship was not seen for individual surgeons.

**Supplementary Information:**

The online version contains supplementary material available at 10.1186/s13019-022-01943-2.

## Background

Referrals for lung transplantation have been increasing with the most common indications being chronic obstructive pulmonary disease (COPD), interstitial lung disease and cystic fibrosis [1]. The immediate effect has been longer waiting lists in view of the persistent shortage of donor lungs suitable for transplantation [2]. This has been partially mitigated by the use of the lung allocation score introduced in 2005 by the Organ Procurement and Transplantation Network (OPTN) in the United States [3] and the National Urgent Lung Allocation Scheme in the United Kingdom (UK) to ensure that patients most in need receive their transplant as quickly as possible.

Appropriate allocation of donor lungs to highest priority patients is still limited due to a number of factors such as ischaemic time. Despite this, it was shown that in larger centres the impact of longer ischaemic time was less significant, with no disadvantage when compared to shorter ischaemic times in the same centre [4]. This indicates that better patient outcomes are associated with management strategies in more experienced units.

There are a number of studies supporting the assertion that larger centres have improved survival rates particularly in complex procedures such as cardiac and paediatric cardiac surgery [5, 6]. A number of contributing factors have been implicated, particularly the standard of peri-operative care and the quality of post-operative care providers [6]. There has been scant literature to reflect the possible effect of individual surgeon age and experience on survival rates.

A recent study compared surgeon age and its correlation with patient survival in oesophageal carcinoma. This showed improved 5-year mortality figures for surgeons between the age of 52 and 56 years with worsening short and long-term mortality rates for surgeons less than 51 and more than 56 years of age after adjustment for confounding factors [7].

No previous studies have been published to show the effect of individual surgeon experience on post-operative outcomes after lung transplant surgery. The objective of this retrospective cohort study is to investigate the influence of individual surgeons on patient outcomes by the analysis of prospectively collected data for a single lung transplant centre.

## Patients and methods

Prospective databases identified all adult patients undergoing isolated lung transplantation at a single UK centre between June 1987 and October 2017. Demographic data, underlying lung pathology and details of surgical procedure, including ischaemic time, were extracted from the prospective data-sets. Mortality data for these patients was acquired from NHS (National Health Service) Spine.

Patients under the age of 18 years and those with no consultant information were excluded from the study. Procedures carried out by multiple consultant surgeons or by surgeons performing less than 10 transplants were also excluded from analysis. All data was retrospectively analysed. No ethical approval was required for this analysis. The distribution of age of surgeon over the period of the study remained consistent and intensive care unit (ICU) management of lung transplant patients was protocol driven with no major changes in management strategies that may have affected patient outcomes over time.

Individual surgeon demographics for 16 consultants performing lung transplant over this time period were obtained from the General Medical Council and Departmental records. The surgeon age at the time of each transplant was calculated and utilised as a reflection of surgeon experience and technical maturity. Statistical analysis was carried out using Student *t*-test, Pearson’s Chi-squared, ANOVA and Logistic Regression from the Analyse-it package for Microsoft Excel and STATA/IC.

The data was further analysed using the number of previous transplants performed by each transplant surgeon at the time of each individual procedure. The Kaplan–Meier method was used for analysis of survival by surgeon lung transplant volume. A Kaplan–Meier curve was analysed for 3 groups according to incident number as follows: the first 20 transplants performed by the surgeon, the next 30 transplants and then all other transplants performed (51 +). Survival difference in these groups has been portrayed by super-imposed Kaplan–Meier curves for each group.

Reporting of this research has been carried out according to Strengthening the Reporting of Observational Studies in Epidemiology (STROBE) guidelines available at www.strobe-statement.org.

## Results

A total of 954 transplants were performed over 30 years in a single UK centre with a median survival to follow-up of 4.37 years. 55.9% of lung transplants performed were male and 54.1% female with an average age of 44.4 ± 13.8 years. 648 transplants (67.9%) were bilateral lung transplants and 306 (32.1%) were single lung transplants.

Patient characteristics according to individual surgeon transplant volume did not differ significantly as indicated in Table 1. Patient age, pathology and mean ischaemic times for donor lungs were not statistically significant (*p* = 0.194, *p* = 0.184 respectively). There was a positive correlation with an increasing percentage of bilateral lung transplants carried out by surgeons with increased lung transplant incident volume (Pearson correlation, *p* = 0.002).

**Table 1:** Patient Characteristics according to surgeon incident volume

Patient characteristics	First 20 transplants	21–50 transplants	> 51 transplants	Statistical test, *p* value
*TOTAL*	*n* = *238*	*n* = *186*	*n* = *355*	
Gender Male	126 (52.9%)	96 (51.6%)	220 (62%)	Pearson correlation, positive correlation *p* = 0.041
Gender Female	112	90	135	
Age (years)	43.6 ± 1.826	45.4 ± 2.048	45.6 ± 1.415	ANOVA, *p* = 0.194
Average Ischaemic Time (minutes)	339.519 ± 12.355	336.783 ± 16.641	324.571 ± 11.314	ANOVA, *p* = 0.184
Single	85	53	80	
Bilateral	153 (64.3%)	133 (71.5%)	275 (77.5%)	Pearson correlation, positive correlation with increasing incident volume, *p* = 0.002

Native lung pathology and indication for transplant is further elaborated in Additional file 1: Table S3.

Similarly, there were no statistically significant differences in patient characteristics operated by surgeons of different age groups as identified in Table 2. There was a positive correlation of increasing surgeon age with an increasing percentage of bilateral lung transplants (Pearson correlation, *p* = 0.0001).

**Table 2:** Patient Characteristics according to surgeon age

Patient characteristics	Surgeon Age < 45 years	Surgeon Age 45 – 50 years	Surgeon Age > 51	Statistical test, *p* value
*TOTAL*	*n* = *207*	*n* = *271*	*n* = *283*	
Gender Male	113 (54.6%)	150 (55.4%)	171 (60.4%)	Pearson correlation, no correlation *p* = 0.259
Gender Female	94	121	112	
Age (years)	44.4 ± 1.966	44.1 ± 1.657	46.4 ± 1.583	ANOVA, *p* = 0.100
Average Ischaemic Time (minutes)	325.856 ± 11.758	329.621 ± 12.013	340.305 ± 14.96	ANOVA, *p* = 0.268
Single	80	64	68	
Bilateral	127 (61.4%)	207 (76.4%)	215 (76%)	Pearson correlation, positive correlation with increasing surgeon age, *p* < 0.0001

**Fig. 1:**
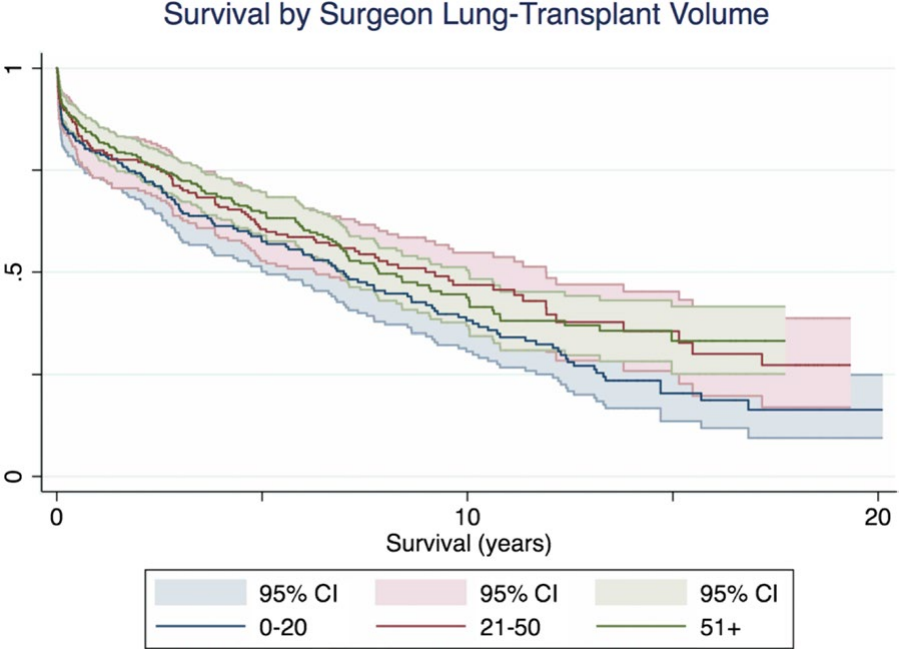
Kaplan–Meier survival curves according to surgeon transplant incident volume, with 20 or less transplants, 21–50 transplants and > 51 transplants performed by the operating surgeon. 95% CI bands for each group indicated. There was no difference in 5-year survival between the individual surgeon experience groups (OR 1.002, 95%CI: 0.999–1.004, *p* = 0.192)

Individual surgeon experience, measured by the number of previous lung transplants performed (mean, 76.4 ± 78 procedures), showed a correlation with improved outcomes at 30 days (OR 1.004, 95%CI: 0.9999 to 1.008, *p* = 0.0413) but there was no difference in 5-year survival (OR 1.002, 95%CI: 0.999—1.004, *p* = 0.192). Figure 1 demonstrates Kaplan–Meier survival curves with 95% CI bands, for three surgeon-experience groups (0–20, 21–50, and > 51 previous lung transplants).

There was no difference in survival by recipient gender (*p* = 0.661) or between individual surgeons (*p* = 0.224). No difference was demonstrated between weekday and weekend procedures (*p* = 0.327).

Increasing total centre experience with lung transplantation (OR 1.001, 95%CI: 1.000–1.001, *p* = 0.03) and with successive calendar years (OR 1.028, 95%CI: 1.005–1.052, *p* = 0.017) was associated with improved 5-year survival.

**Fig. 2:**
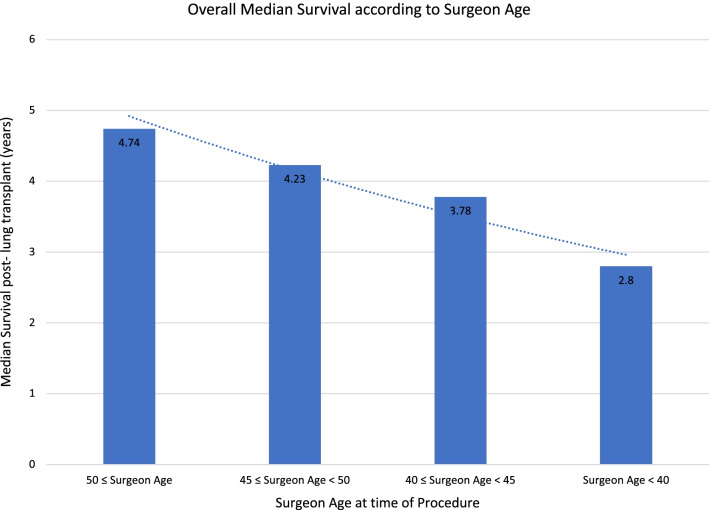
Overall median survival according to surgeon age

Advancing surgeon age at the time of performing the transplant (mean, 48.8 ± 6.6 years) was associated with an improved 30-day survival (OR 1.062, 95%CI: 1.019 to 1.106, *p* = 0.003), which persisted at 5 years post-transplant (OR 1.043, 95%CI: 1.014–1.073, *p* = 0.003). Figure 2 depicts a decrease in median overall survival with decreasing surgeon age.

## Discussion

Patient outcomes and mortality after surgery are the focus of multiple studies which have guided progress, the development of new protocols and in-hospital management. There appears to be a definite positive relationship between larger hospital case volumes and improved patient survival, particularly in more complex procedures [5, 8, 9]. Despite this, there have been few studies to examine the effect of the individual surgeon’s experience or age on patient mortality. In high volume hospitals, there are a number of factors that could contribute to improved survival, including better post-operative care, resources, technology and staffing [10].

A study based in the United States attempted to identify the relationship of surgeon experience in high volume centres. It found that in procedures such as open heart surgery and pancreatic resections, a significant percentage of improved survival was attributable to individual surgeon volume thus implying that surgeon experience has a crucial role in improved patient outcomes [10].

In thyroidectomy, looking specifically at the effect of a higher volume of cases per surgeon compared to patient outcome, a lower complication rate (*p* < 0.001) and shorter hospital stay in higher volume surgeons was demonstrated. More experienced surgeons were seen to take more complicated cases and this was corrected for in the results [11].

A Taiwanese study which included almost 10,000 coronary artery bypass grafting (CABG) operations performed by 316 surgeons found a significantly higher mortality comparing low volume surgeons with high volume surgeons (*p* < 0.001). The same could not be said for hospital volumes (*p* = 0.078), with the interesting conclusion that surgeon experience has a more significant effect on patient outcome than hospital volume [12]. This supported an older study with positive surgeon volume outcomes for CABG [13].

The age groups of 244 surgeons were analysed examining three surgeon age groups (< 40 years, 40 – 45 years and > 45 years of age) and found a significant difference in patient mortality after coronary artery bypass grafting, with older surgeons having decreased in-hospital mortality rates (*p* < 0.05). A similar trend was noted with surgeons having an increased caseload (*p* = 0.038) [14]. This compares favourably with our results which also identified a trend towards improved patient survival in surgeons with a higher lung transplant caseload, although this was not significant after 5 years.

For transplant patients, the acceptance of organs also plays a role. Reports have shown that the UK has a relatively high acceptance rate of around 45% in 2012–2013 and 47% in 2016–2017, with our centre having an acceptance rate above the UK average, except during the period 2015–2016 [15, 16]. This compares to a 24% acceptance rate for lung organ donations in the United States for the years 2016–2017 [17]. This acceptance rate tends to decline on second and third offers of the same organs. Furthermore, acceptance at a different centre may mean that the organ may need to travel further with a potentially longer ischaemic time [17]. The decision-making involved in the assessment of the organs offered, on balance with known waiting list mortality rates, particularly for high risk patients such as those suffering from cystic fibrosis, may improve with more experience in the field. Surgeons with more experience may be more or less likely to refuse borderline organs and this would be an interesting aspect to explore with analysis on effect of patient survival. Unfortunately, we did not have the data for declined organs per consultant surgeon in our centre for comparison purposes.

In our results we noted a significant trend for increasing number of bilateral lung transplant cases, as compared to single lung transplant, in older surgeons. Literature has shown that bilateral lung transplant may have a slight survival advantage overall [18, 19]. The underlying reason for this remains unclear [18], especially in the face of the different disease processes involved [19]. It is unclear why older surgeons in our centre did more bilateral lung transplants overall and this may be secondary to the increasing surgical confidence that comes with experience; however, we acknowledge that this may have contributed to improved survival in this group.

An alternate aspect of a surgeons practice is patient follow up post-surgery. This was analysed from a lung cancer perspective by Johnson et al*.*, and no significant difference was found in the management of patients after lung cancer resections across surgeon age groups [20]. Management was uniformly in compliance with the accepted and most recent guidelines highlighting a high level of continuous professional development throughout a surgeon’s profession [20]. A similar result was found for colorectal surgeons in the United States in the follow up of colorectal carcinoma [21]. Of note, both these studies were carried out by the use of a questionnaire filled by the practicing surgeon with a less than 40% response rate.

With ongoing advances in the post-operative management after lung transplantation, this would be an interesting subject to explore further, particularly in view of the management by a physician rather than a surgeon based team in the long term. A physician based study of doctors managing acute myocardial infarction showed that a volume relationship was also significant with physicians, showing a 10% decrease in mortality with every 16 extra cases treated by the physician (*p* = 0.05). Contrary to the effect described above; physicians in this study were seen to have increasing mortality when related to years since graduation from medical school [22].

The effect of experience on surgical outcomes has had a significant effect on surgical training. The growing focus on patient outcomes, time efficiency and expectation of the attending surgeon being present throughout surgery has made it harder for trainees to develop autonomy in the practice of surgery and patient management. The impact of regulated hours and a move towards a shift-style of working has also been implicated in trainees feeling inexperienced at the end of their training [23]. Naturally, there is recognition of poorer patient outcomes for complex surgeries carried out by younger surgeons; although similar patient outcomes were identified for less complex procedures [24].

Increasing age is associated with a general deterioration in physical attributes and percipience found to be key factors in a surgeons’ skill [25]. Standard operating procedures utilised to fly aircraft, particularly commercial aircraft, have often been used to develop training and checklists in surgical theatres [26]. However, pilots are forced to retire at the age of 65 according to European Union regulations and those in the United States. In comparison, there is no definite age for retirement in surgeons and many do not plan for retirement [27].

It appears that despite the expected decline in visual acuity and dexterity with age, experience may often compensate for this loss in the surgical field [25]. On the other hand, a study in general surgery investigating hernia repair recurrence rates showed that surgeons above the age of 45 years had worse results than younger surgeons with the same experience [28], indicating that experience and age contribute to different extents to improved post-surgical outcomes.

Although our study has shown an improvement of patient outcomes with increasing surgeon age, this may not be expected to increase exponentially. This was emphasised in oesophagectomies for carcinoma, where despite a clear improvement in all-cause and disease-specific 5-year mortality with increasing surgeon age, this was only illustrated up to the surgeon age of 56 years, after which an increase in mortality rates was noted [7].

### Limitations

This study is limited to a single centre and it would be interesting to expand this study to other lung transplant centres to compare individual surgeon results, as well as assess patient volume in different centres and the reusltant effect on outcomes. Patients in our study were treated over a time-period of 30 years, with the effect of improving patient management from an intensive care perspective, patient selection and improved retrieval and operative techniques, being difficult to estimate in our results due to the multi-factorial nature of these improvements. In this regard, further studies encompassing transplant outcomes over the past 10 years may further highlight current protocols for lung transplant management and their effect on outcomes.

## Conclusion

In conclusion, it is evident that in lung transplantation, surgeon age contributes significantly to post-surgical patient outcomes in our study. There is a trend within the literature to support a correlation between increased surgeon experience and volume, and improved patient mortality. Investigation in larger surgeon cohorts and across multiple centres will help to further identify emerging trends. Further study will be necessary to identify surgeon-specific factors contributing to this effect. This will help to develop training programmes and ensure adequate continuous professional development to ensure high quality of care throughout the patients’ surgical journey.

## Supplementary Information

**Additional file 1: Table S3 **Lung pathology incidence for our dataset

## Data Availability

The datasets used and/or analysed during the current study are available from the corresponding author on reasonable request.

## Abbreviations

CI: Confidence interval
COPD: Chronic obstructive pulmonary disease
ICU: Intensive care unit
NHS: National health service
OPTN: Organ procurement and transplantation network
OR: Odds ratio
UK: United Kingdom
