# Supplementary material for: Do older surgeons have safer hands? A retrospective cohort study
Source: J Cardiothorac Surg. 2022 Sep 1;17:223. doi: 10.1186/s13019-022-01943-2 (PMC9438167; doi:10.1186/s13019-022-01943-2)
Supplement: Supplementary file 1 — Additional file 1: Table S3 Lung pathology incidence for our dataset [file 13019_2022_1943_MOESM1_ESM.pdf]

Supplementary Table 3		
**Pathology**	n = 954	%
Alpha 1 Antitrypsin Deficiency	37	3.88
Asthma	12	1.26
Bronchiectasis	50	5.24
COPD	78	8.18
Cystic Fibrosis	303	31.76
Emphysema	151	15.83
Fibrosing Alveolitis	57	5.97
Fibrotic lung disease	72	7.55
Histiocytosis X	11	1.15
Idiopathic Pulmonary Fibrosis	46	4.82
Interstitial Lung Disease	34	3.56
Lymphangioleiomyomatosis	20	2.10
Obliterative Bronchiolitis	21	2.20
Other Lung Disease	10	1.05
Primary pulmonary hypertension	12	1.26
Pulmonary Fibrosis	16	1.68
Pulmonary Hypertension	5	0.52
Sarcoidosis	19	1.99
